# Ser46 phosphorylation of p53 is an essential event in prolyl-isomerase Pin1-mediated p53-independent apoptosis in response to heat stress

**DOI:** 10.1038/s41419-019-1316-8

**Published:** 2019-02-04

**Authors:** Li Li, Zijun Su, Zhimin Zou, Hongping Tan, Daozhang Cai, Lei Su, Zhengtao Gu

**Affiliations:** 1 Emergency & Intensive Care Unit, The Third Affiliated Hospital, Southern Medical University, Guangzhou, China; 2 Academy of Orthopedics Guangdong Province, Guangzhou, China; 3 Department of Pathophysiology, Southern Medical University, Guangdong Provincial Key Laboratory of Shock and Microcirculation Research, Guangzhou, China; 4 Department of Pathology, Lawson Health Research Institute, University of Western Ontario, London, Ontario, Canada; 5 Department of Epilepsy Surgery, Guangdong Sanjiu Brain Hospital, Guangzhou, China; 6 Department of Orthopedics, The Third Affiliated Hospital, Southern Medical University, Guangzhou, China; 7 Department of Intensive Care Unit, Guangzhou General Hospital of Guangzhou Military Command, Key Laboratory of Tropical Zone Trauma Care and Tissue Repair of PLA, Guangzhou, China; 8 Department of Treatment Center For Traumatic Injuries, The Third Affiliated Hospital, Southern Medical University, Guangzhou, China

## Abstract

Heat stroke has increased in frequency worldwide in recent years and continues to have a high morbidity and mortality. Identification of the mechanisms mediating heat stoke is important and necessary. Our preliminary study revealed heat stress (HS)-induced apoptosis of vascular endothelial cells was associated with reactive oxygen species (ROS)-induced p53 translocation into mitochondria. Previous studies have suggested the prolyl-isomerase Pin1 regulates p53 functioning through specific binding to p53 phosphorylation sites. Based on these studies, we presumed Pin1 is a key intermediate in regulation of mitochondrial p53 translocation through a HS-induced ROS-p53 transcription-independent apoptosis pathway. In this context, we revealed p53 had a crucial role in a HS-induced mitochondrial apoptotic pathway, where p53 protein rapidly translocated into mitochondria in endothelial cells both in vitro and in vivo. In particular, HS caused an increase in p53 phosphorylation at Ser46 that facilitated interactions with phosphorylation-dependent prolyl-isomerase Pin1, which has a key role in promoting HS-induced localization of p53 to mitochondria. Furthermore, we also found ROS production was a critical mediator in HS-induced Pin1/p53 signaling and was involved in regulating mitochondrial apoptosis pathway activation. Therefore, we have contributed to our profound understanding of the mechanism underlying HS-induced endothelial dysfunction in an effort to reduce the mortality and morbidity of heat stroke.

## Introduction

The intensity, frequency, and duration of heat waves have increased, especially over the past decades due to the changing climate and, therefore, it is feared that the number of patients with heat-associated illnesses may continue to increase^1–3^. One severe life-threatening heat-associated illness is heat stroke, which is clinically considered to be when the core body temperature increases to above 40 °C and is often associated with hot, dry skin, and abnormalities of the central nervous system^4^. Despite several decades of research, heat stroke continues to cause high incidences of morbidity, mortality, and multiple organ dysfunction syndromes (MODSs) in patients^5,6^. Furthermore, there is a limited understanding of the mechanisms mediating MODS during heat stroke. Therefore, it is important to investigate the pathogenesis of heat stroke and develop effective preventive and treatment methods accordingly. Studies using cell lines and animal models found vascular endothelial cells are early targets of heat stress (HS) injury^5–7^ and further research revealed apoptosis of vascular endothelial cells is a prominent feature of heat stroke^8–10^. Therefore, apoptosis of vascular endothelial cells appears to be involved in heat stroke pathogenesis, although the associated mechanisms need to be further delineated.

The protein p53 regulates a number of pathways, including those involved in energy metabolism, genomic stability, antioxidant functions, and DNA damage, and promotes either cytostatic or cytotoxic responses following exposure to exogenous or intrinsic cellular stress^11^. Due to the complexity of the intracellular functions of p53, a deeper understanding of the convergence of signaling networks at this hub mediating HS-dependent toxicity is needed to characterize the reduction in vascular endothelial cell survival during HS. We previously demonstrated that reactive oxygen species (ROS) are involved in the signaling events that lead to mitochondrial translocation of p53 in human umbilical vein endothelial cells (HUVECs)^9,10^. Oxidative stress is also thought to play a pivotal role in HS-induced apoptosis of HUVECs^4,9,10^. Our work indicates that, during HS-induced apoptosis of HUVECs, mitochondrial translocation of p53 is involved in triggering of ROS-dependent apoptosis. However, the precise mechanism by which HS leads to apoptosis of vascular endothelial cells remains largely unclear.

Pin1 is a highly conserved peptidyl-prolyl cis/trans isomerase that specifically recognizes phosphorylated Ser/Thr-Pro peptide bonds and induces conformational changes with high efficiency in its substrates^12–14^. This Pin1-catalyzed isomerization changes the activity of many phosphoproteins, thus controlling a number of signaling pathways involved in a variety of activities, including gene transcription, tumor development, redox balance, and apoptosis^13–15^. In the face of genotoxic insults, Pin1 binds to multiple sites on p53, including the phosphorylation sites Ser33, Ser46, Thr81, and Ser315^16–20^. This promotes p53 dissociation from HDM2, which causes consequent accumulation in stressed cells, and the apoptosis inhibitor inhibitory member of the apoptosis stimulating protein of p53 family (iASPP), which works through isomerization of the phospho-Ser46-Pro47 motif, thus unleashing the full apoptotic potential of p53^17,19,21^. However, Pin1 isomerization control of p53 functioning through alterations in sub-cellular trafficking has never been assessed in HS-induced damage to vascular endothelial cells.

In the present study, we characterized the mechanisms involved in p53 promotion of the direct mitochondrial death program. Specifically, we demonstrated a crucial role for Pin1 involvement in the ROS-p53 route of apoptosis triggered in response to HS in vascular endothelial cells.

## Results

### Localization of p53 to the mitochondria played an essential role in mediation of HS-induced apoptosis

**Fig. 1:**
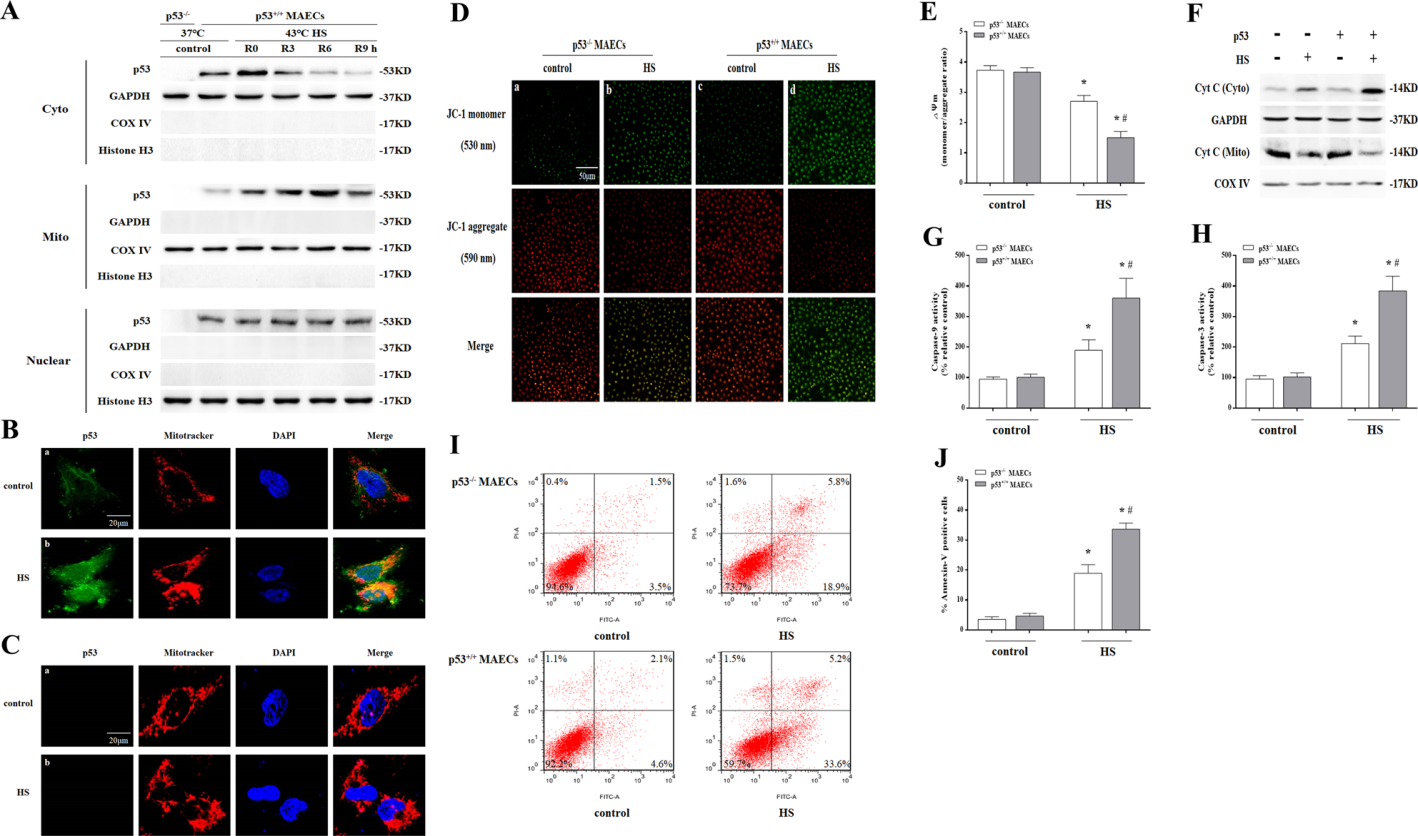
Localization of p53 to the mitochondria played an essential role in mediating heat stress (HS)-induced apoptosis in *p53*^+/+^ mouse aortic endothelial cells (MAECs). Both *p53*^+/+^ and *p53*^-/-^ MAECs were isolated from wild-type and *p53*^KO^ mice, respectively. **a** The *p53*^+/+^ MAECs were exposed to intense HS (43 °C) for 2 h and then incubated at 37 °C for the indicated durations of 0, 3, 6, or 9 h. The levels of p53 protein in the mitochondrial, cytosolic, and the nuclear fractions were assessed by western blot. GAPDH, cytosolic loading control; COX IV, mitochondrial loading control; Histone H3, nuclear loading control. The *p53*^-/-^ and *p53*^+/+^ MAECs were exposed to 43 °C for 2 h and then incubated at 37 °C for 6 h (**b**–**j**). **b**–**c** Confocal laser scanning microscopy revealed localization of p53 to the mitochondria upon HS. Green fluorescence represents p53, red fluorescence represents Mito Tracker, and orange fluorescence represents translocation of p53 from the cytoplasm to the mitochondria. **d** Confocal laser scanning microscopy revealed localization of JC-1 aggregates and monomers in the cells. Green fluorescence represents JC-1 monomers and red fluorescence represents JC-1 aggregates. **e** Quantification of mitochondrial membrane potential changes (JC-1 monomer/JC-1 aggregates). **f** Cyt C protein levels in the mitochondrial and cytosolic fractions were assessed by western blot. **g** Enzymatic activity of Caspase-9 was measured using fluorogenic substrate Ac-LEHD-AFC. **h** Enzymatic activity of Caspase-3 was measured using fluorogenic substrate Ac-DEVD-AMC. **i** Apoptosis was analyzed by flow cytometry using Annexin V-FITC/PI staining. **j** Quantification of apoptosis induced by HS. ^*^P < 0.05 compared with the control group (37 °C); ^#^*P*  < 0.05 compared with the HS group of *p53*^-/-^ MAECs

**Fig. 2:**
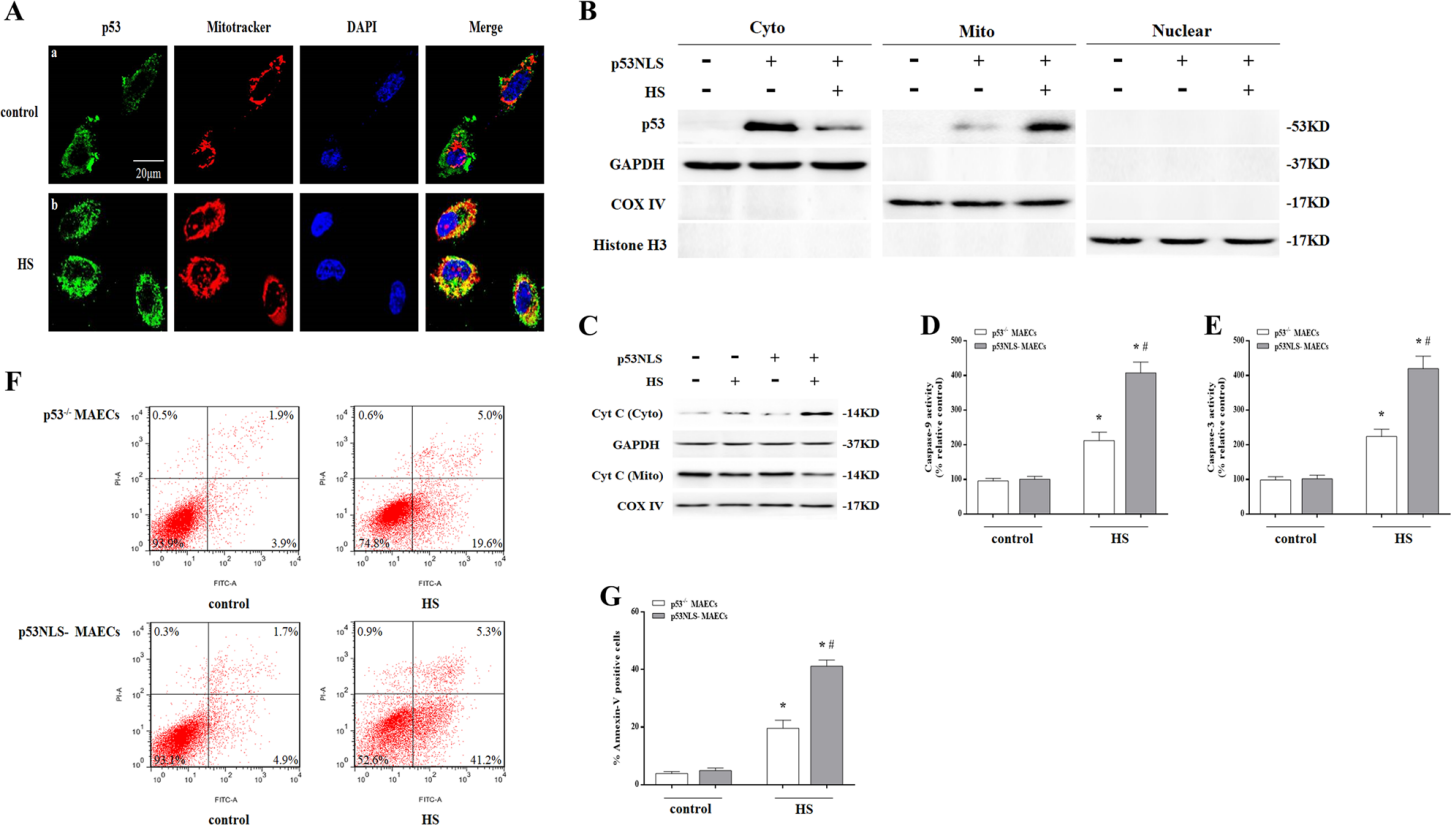
Heat stress (HS)-induced p53 mitochondrial localization-mediated mitochondrial apoptosis pathway in p53 NLS- mouse aortic endothelial cells (MAECs). A nuclear import-deficient p53 construct (p53NLS-) was introduced into *p53*^-/-^ MAECs. Then *p53*^-/-^ and p53NLS- MAECs were exposed to 43 °C for 2 h and further incubated at 37 °C for 6 h. **a** Confocal laser scanning microscopy revealed localization of p53 to the mitochondria in cells in response to HS. Green fluorescence represents p53, red fluorescence represents Mito Tracker, and orange fluorescence represents translocation of p53 from the cytoplasm to the mitochondria. **b** The levels of p53 protein in the mitochondrial, cytosolic and the nuclear fractions were assessed by western blot. GAPDH, cytosolic loading control; COX IV, mitochondrial loading control; Histone H3, nuclear loading control. **c** The levels of Cyt C in the mitochondrial and cytosolic fractions proteins were assessed by western blot. **d** Enzymatic activity of Caspase-9 was measured using fluorogenic substrate Ac-LEHD-AFC. **e** Enzymatic activity of Caspase-3 is measured using fluorogenic substrate Ac-DEVD-AMC. **f** Apoptosis was analyzed by flow cytometry using Annexin V-FITC/PI staining. **g** Quantification of apoptosis induced by HS. ^*^P < 0.05 compared with the control group (37 °C); ^#^*P* < 0.05, compared with the HS *p53*^-/-^ MAEC group

We isolated aortic endothelial cells from wild-type and *p53* gene knockout (*p53*^KO^) mice and isolated *p53*^+/+^ and *p53*^-/-^ mouse aortic endothelial cells (MAECs), respectively. As shown in Fig. 1a, we isolated the nuclear, cytosolic, and mitochondrial fractions from MAECs, and exposure of *p53*^+/+^ MAECs to HS for 2 h and further incubation for different time periods resulted in a significant amount of p53 translocation in the mitochondria in a time-dependent manner was evident at 3 h and peaking at 6 h, whereas there was no difference in the nuclear p53 between control and HS group in *p53*^+/+^ MAECs. Immunofluorescence was used to directly observe localization of p53 in mitochondria. It was found p53 (green) and Mito Tracker staining (red) overlapped at 6 h after HS in *p53*^+/+^ MAECs (Fig. 1b(b)). There was no p53 signal in *p53*^-/-^ MAECs (Fig. 1c). Further studies found HS resulted in a reduction in mitochondrial membrane potential (Fig. 1d(d) and e) and promoted release of cytochrome c (Cyt C) from mitochondria (Fig. 1f), activation of Caspase-9/-3 (Fig. 1g, h), and induced apoptosis by 33.6% (Fig. 1i, j) in *p53*^+/+^ MAECs; whereas *p53*^-/-^ MAECs has obvious prevention effect on HS-induced mitochondrial membrane potential loss and Cyt C release, Caspase-9/-3 activity, and apoptosis (Fig. 1d–j). To verify p53 mitochondrial translocation as a major step in HS-induced apoptosis, we employed a nuclear import-deficient p53 construct (p53NLS-) in *p53*^-/-^ MAECs (p53NLS- MAECs), which was no p53 expression in the nucleus of MAECs (Fig. 2a, b). As shown in Fig. 2a, b, HS led to a significant amount of p53 accumulation in the mitochondria of p53NLS- MAECs. Furthermore, p53NLS efficiently induced Cyt C release, Caspase-9/-3 activity, and apoptosis in p53NLS- MAECs treated with HS (Fig. 2c–g).

**Fig. 3:**
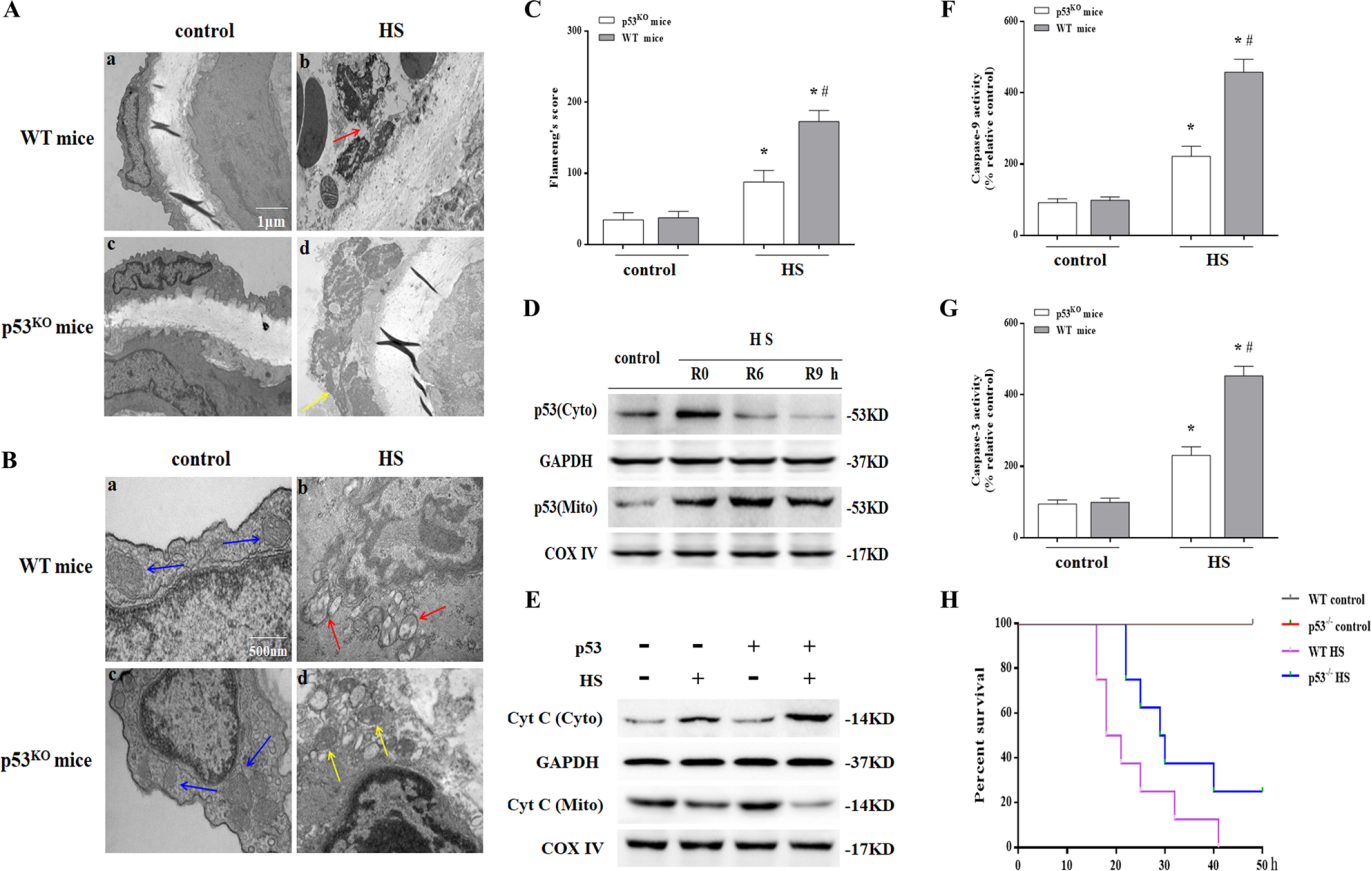
Heat stress (HS)-induced p53 mitochondrial localization-mediated apoptosis in aortic endothelium. The animals in the control group were sham heated at a temperature of 25 ± 0.5 °C and a humidity of 35 ± 5% for a time comparable to that of the HS group. The animals in the HS group were placed in a temperature-controlled chamber (ambient temperature 35.5 ± 0.5 °C and 60 ± 5% relative humidity) and their rectal core temperature (Tc) was continuously monitored with a rectal thermometer until the Tc reached 42 °C. The mice were sacrificed 6 h after HS and the aortic endothelium was isolated. **a** Aortic endothelium morphology of was observed by transmission electron microscopy (TEM). **a**(a) and **a**(c) represent the control group in wild-type and *p53*^KO^ mice, respectively; **a**(b) represents the HS group in wild-type mice, the red arrow indicates damaged endothelial cells; **a**(d) represents the HS group in *p53*^KO^ mice, the yellow arrow indicates damaged endothelial cells. **b** Mitochondrial morphology in endothelial cells of the aorta was observed by TEM. **b**(a) and **b**(c) represent the control group in wild-type and *p53*^KO^ mice, respectively, where mitochondrial morphology was normal with preserved membranes and cristae (blue arrows); **b**(b) represents the HS group in wild-type mice, where the mitochondria were severely damaged, appeared swollen, were irregularly shaped, and had disrupted and poorly defined cristae (red arrows); **b**(d) represents the HS group in *p53*^KO^ mice, where the mitochondria were partially damaged, appeared mildly swollen and irregularly shaped, and less damaged than in wild-type mice (yellow arrows). **c**. Mitochondria structural damage was evaluated by Flameng’s score. **d**. The mice were sacrificed at 0, 6, and 9 h after HS and the aortic endothelium was isolated. The levels of p53 in the mitochondrial and cytosolic fractions were assessed by western blot. **e** The levels of Cyt C in the mitochondrial and cytosolic fractions were assessed by western blot. **f** Enzymatic activity of Caspase-9 was measured using fluorogenic substrate Ac-LEHD-AFC. **g** Enzymatic activity of Caspase-3 was measured using fluorogenic substrate Ac-DEVD-AMC. **h** Kaplan–Meier program for survival duration of mice in each group. ^*^P < 0.05 compared with the control group (37 °C); ^#^*P* < 0.05 compared with the HS group in *p53*^KO^ mice, *n* = 6

Meanwhile, we isolated aortic endothelium from wild-type and *p53* ^KO^ mice and assessed the structure of MAECs with transmission electron microscopy (TEM). As shown in Fig. 3a(a) and a(c), MAECs from wild-type or *p53* ^KO^ mice in the control group had normal structures. However, the structures of the aortic endothelium in the HS group displayed remarkable morphological abnormalities, where most endothelial cells were severely swollen, resulting in a rough cell surface (Fig. 3a(b)). In addition, as shown in the HS group (Fig. 3b(b) and c), most mitochondria in the endothelial cells were swollen and irregularly shaped with disrupted and poorly defined cristae. This endothelial histological injury was strikingly absent in *p53* ^KO^ mice (Fig. 3a(d), b(d) and c). HS also induced p53 translocation in the mitochondria, promoted Cyt C release, and activated Caspase-9/-3 in aortic endothelium (Fig. 3d–g), whereas cells deficient in p53 lacked inducement of the mitochondrial apoptosis pathway in aortic endothelium by HS (Fig. 3e–g). Furthermore, the overall survival rate in the *p53* ^KO^ mice HS group was significantly higher than in the wild-type HS group (Fig. 3h). Collectively, these findings indicate HS-induced p53 mitochondrial translocation that promoted mitochondrial apoptosis pathway activation both in vitro and in vivo and deletion of the *p53* gene alleviated endothelial cell injury and increased the overall survival rate.

### HS stimulated p53 activation and interactions with Pin1

**Fig. 4:**
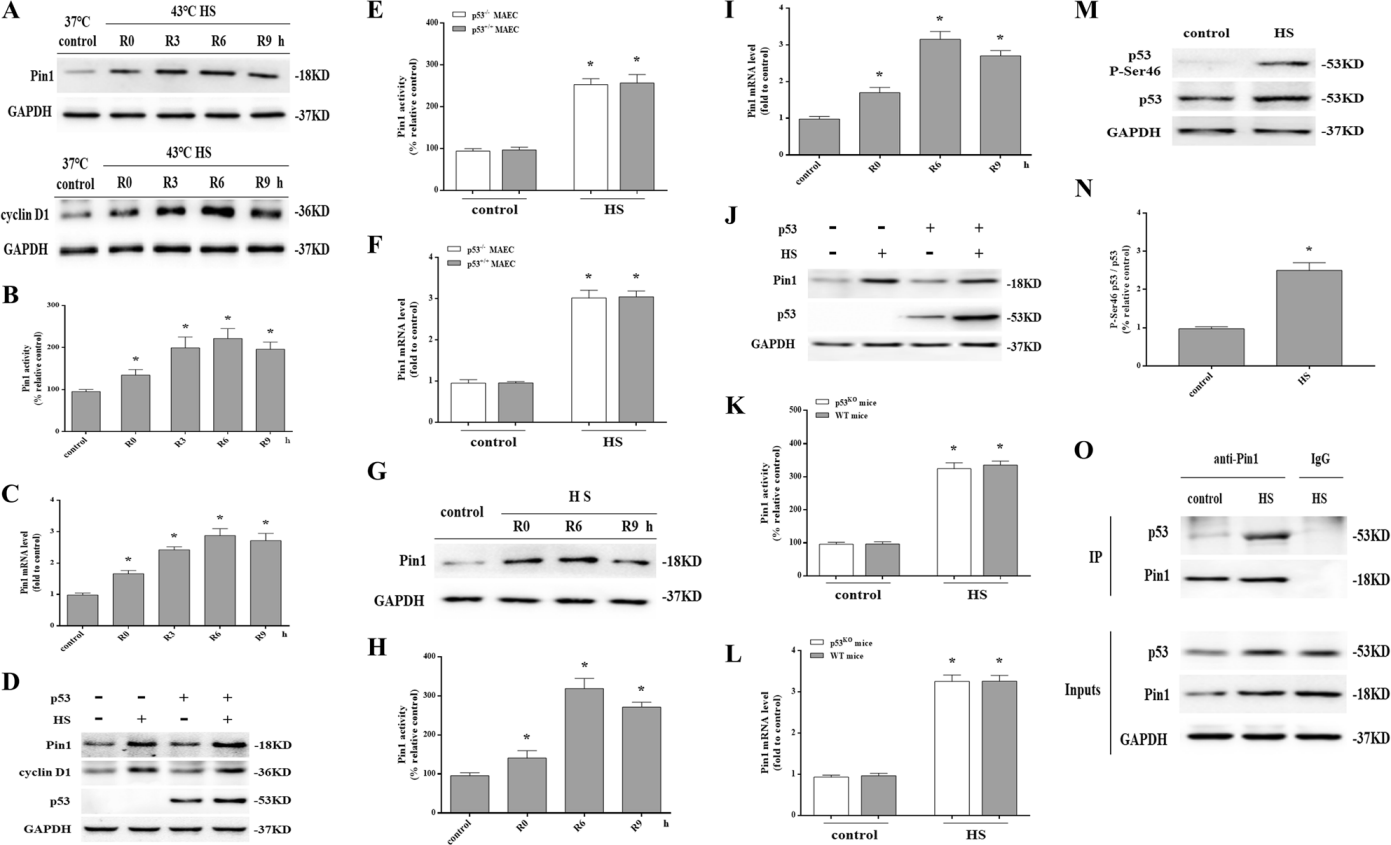
Pin1 expression was increased by heat stress (HS) and required for efficient mitochondrial localization of p53. The *p53*^+/+^ mouse aortic endothelial cells (MAECs) were subjected to intense HS (43 °C) for 2 h and then further incubated at 37 °C for 0, 3, 6, or 9 h (**a**–**b**). **a** Pin1 and cyclin D1 protein levels were assessed by western blot. **b** Quantification of Pin1 activity at the indicated time points after HS. **c** Quantification of Pin1 mRNA at the indicated time points after HS. The *p53*^-/-^ and *p53*^+/+^ MAECs were exposed to 43 °C for 2 h and then further incubated at 37 °C for 6 h (**d**–**f**). **d** Pin1 and p53 protein levels were assessed by western blot. **e** Quantification of Pin1 activity induced by HS. **f** Quantification of Pin1 mRNA induced by HS. Mice were put through the same HS assay as previously described. The mice were sacrificed at 0, 6, and 9 h after exposure to HS and the aortic endothelium was isolated (**g**–**i**). **g** Pin1 protein levels were measured by western blot. **h** Quantification of Pin1 activity at the indicated time points after HS. **i** Quantification of Pin1 mRNA at the indicated time points after HS. Mice were put through the same HS assay as previously described. The mice were sacrificed at 6 h after exposure to HS and the aortic endothelium was isolated (**j**–**l**). **j** Pin1 protein levels were measured by western blot. **k** Quantification of Pin1 activity induced by HS. **l** Quantification of Pin1 mRNA induced by HS. Human umbilical vein endothelial cells (HUVECs) were exposed to intense heat (43 °C) for 2 h and then incubated at 37 °C for 6 h (**m**–**o**). **m** Expression of P-Ser 46 p53 and p53 were determined by western blot. **n** Quantification of the P-Ser 46 p53 and p53 ratio. **o** The *p53*^+/+^ MAECs were exposed to 43 °C for 2 h and were further incubated at 37 °C for 6 h. Cell lysates were normalized for p53 protein levels and then subjected to co-immunoprecipitation to analyze interactions between endogenous p53 and Pin1. IgG served as the negative control. ^*^P < 0.05 compared with the control group (37 °C) in *p53*^+/+^ MAECs or the control group in wild-type mice, *n* = 6

To investigate whether HS affects Pin1 protein expression, *p53*^+/+^ MAECs were subjected to 2 h of HS and then incubated at 37 °C for another 0, 3, 6, or 9 h. Compared with the control group, a significant induction of Pin1 expression and Pin1’s substrates cyclin D1 were observed after 0–9 h of incubation (Fig. 4a). HS also increased Pin1 enzymatic activity (Fig. 4b) and Pin1 mRNA level (Fig. 4c). Moreover, there was no difference in Pin1 protein expression, enzymatic activity, and mRNA level between *p53*^+/+^ and *p53*^-/-^ MAECs after HS (Fig. 4d–f). Further in vivo studies showed that HS increased Pin1 protein expression, enzymatic activity, and mRNA level in a time-dependent manner in aortic endothelium with no difference between wild-type and *p53*^KO^ mice (Fig. 4g–l).

Because p53 Ser46 phosphorylation generates a target site for Pin1^19,20^, we next analyzed p53 phosphorylation. As shown in Fig. 4m, n, p53 was phosphorylated on Ser46 in HUVECs after HS. We next performed co-immunoprecipitation experiments to evaluate whether there are direct interaction between Pin1 and p53. As shown in Fig. 4o, HS increased binding of the Pin1 protein to p53 in *p53*^+/+^ MAECs. Therefore, these results show activation of Pin1 and enhanced interaction between Pin1 and p53 occurred in response to HS, which may be related to promotion of p53 mitochondrial translocation.

### Pin1 potentiated p53 transcription-independent apoptotic activity

**Fig. 5:**
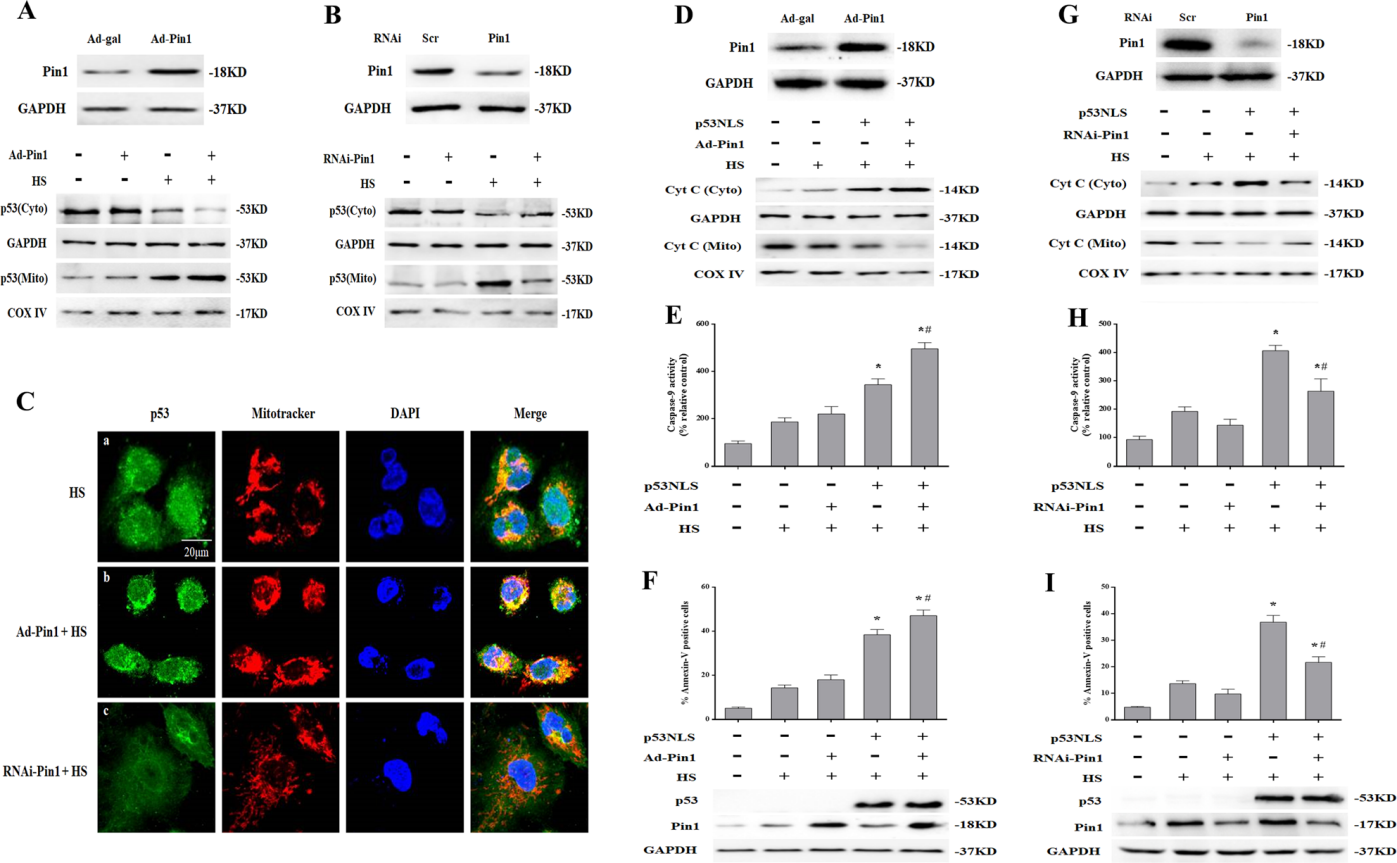
Pin1 potentiated p53 transcription-independent apoptotic activity after exposure to heat stress (HS). The *p53*^+/+^ mouse aortic endothelial cells (MAECs) were transfected with Ad-Pin1 24 h or Pin1 siRNA 48 h in advance, exposed to 43 °C for 2 h, and then further incubated at 37 °C for 6 h (**a**–**c**). **a** Western blots of Pin1 protein expression in transfected cells and intracellular location of p53 in Pin1-overexpressing *p53*^+/+^ MAECs following HS. **b** Western blots of Pin1 protein expression in transfected cells and intracellular location of p53 was determined by western blots in Pin1 RNAi transfected *p53*^+/+^ MAECs following exposure to HS. **c** Confocal laser scanning microscopy assessment of localization of p53 to mitochondria in Pin1-overexpressing or Pin1 RNAi transfected *p53*^+/+^ MAECs exposed to HS. The green fluorescence represents p53, red fluorescence represents Mito Tracker, and orange fluorescence represents translocation of p53 from cytoplasm to mitochondria. H1299 cells were transfected with the p53NLS- construct 48 h and/or Ad-Pin1 24 h in advance. The transfected cells were exposed to 43 °C for 2 h and then further incubated at 37 °C for 6 h (**d**–**f**). **d** Western blots of Pin1 protein expression in transfected cells and the levels of Cyt C in the mitochondrial and cytosolic fractions in Pin1-overexpressing *p53*^+/+^ MAECs following HS. **e** The enzymatic activity of Caspase-9 was measured using fluorogenic substrate Ac-LEHD-AFC. **f** To quantify induction of apoptosis by HS, Annexin V-FITC/PI staining and flow cytometry were used. Western blots of Pin1 and p53 protein expression in transfected cells. H1299 cells were transfected with the p53NLS- construct and/or Pin1 siRNA 48 h in advance. Cells were exposed to 43 °C for 2 h and then incubated at 37 °C for 6 h (**g**–**i**). **g** Western blots of Pin1 protein expression in transfected cells and the levels of Cyt C in the mitochondrial and cytosolic fractions in Pin1 RNAi transfected *p53*^+/+^ MAECs following exposure to HS. **h** Enzymatic activity of Caspase-9 was measured using fluorogenic substrate Ac-LEHD-AFC. **i** Induction of apoptosis in response to HS was analyzed by flow cytometry with Annexin V-FITC/PI staining. Western blots of Pin1 and p53 protein expressed in transfected cells. ^*^P *<* 0.05 compared with the HS group in H1299 *p53*^-/-^ cells (37 °C); ^#^*P* < 0.05 compared with the HS group in H1299 p53NLS- cells

Cytoplasmic p53 translocation to mitochondria is a key step during induction of transcription-independent apoptosis in response to apoptotic stimuli^22,23^. Therefore, we explored whether Pin1 affects mitochondrial localization of p53 in response to HS. Notably, as shown by western blot in Fig. 5a, b, mitochondrial translocation of p53 in HS-treated *p53*^+/+^ MAECs was strongly increased by Pin1 overexpression and reduced by RNA interference (RNAi) knockdown of Pin1. Immunofluorescence staining showed Pin1 overexpression promoted overlap of p53 (green) and Mito Tracker staining (red), whereas knockdown of Pin1 inhibited this phenomenon in HS-treated *p53*^+/+^ MAECs (Fig. 5c).

Since our previous studies have shown HS promotes mitochondrial apoptosis pathway activation in *p53*-deficient cancer cells (H1299 *p53*^-/-^ cells)^9^, further studies investigating the underlying mechanisms of Pin1 and p53 transcription-independent apoptotic activity were performed in H1299 *p53*^-/-^ cells. We employed a nuclear import-deficient p53 construct (p53NLS-) in H1299 *p53*^-/-^ cells and then assessed the effect of Pin1 on the ability of p53NLS- to exert transcription-independent apoptosis in response to HS. As shown in Fig. 5d–f, overexpression of Pin1 promoted HS-induced mitochondrial apoptotic pathway activation, which mainly manifested as Cyt C release from mitochondria to the cytoplasm, Caspase-9 activation, and apoptosis in H1299 p53NLS- cells. Notably, this HS-induced mitochondrial apoptotic pathway activation was inhibited by Pin1 knockdown in H1299 p53NLS- cells (Fig. 5g–i). Interestingly, Pin1 overexpression and knockdown had no effect on mitochondrial apoptotic pathway activation in H1299 *p53*^-/-^ cells after HS (Fig. 5e, f, h and i).

**Fig. 6:**
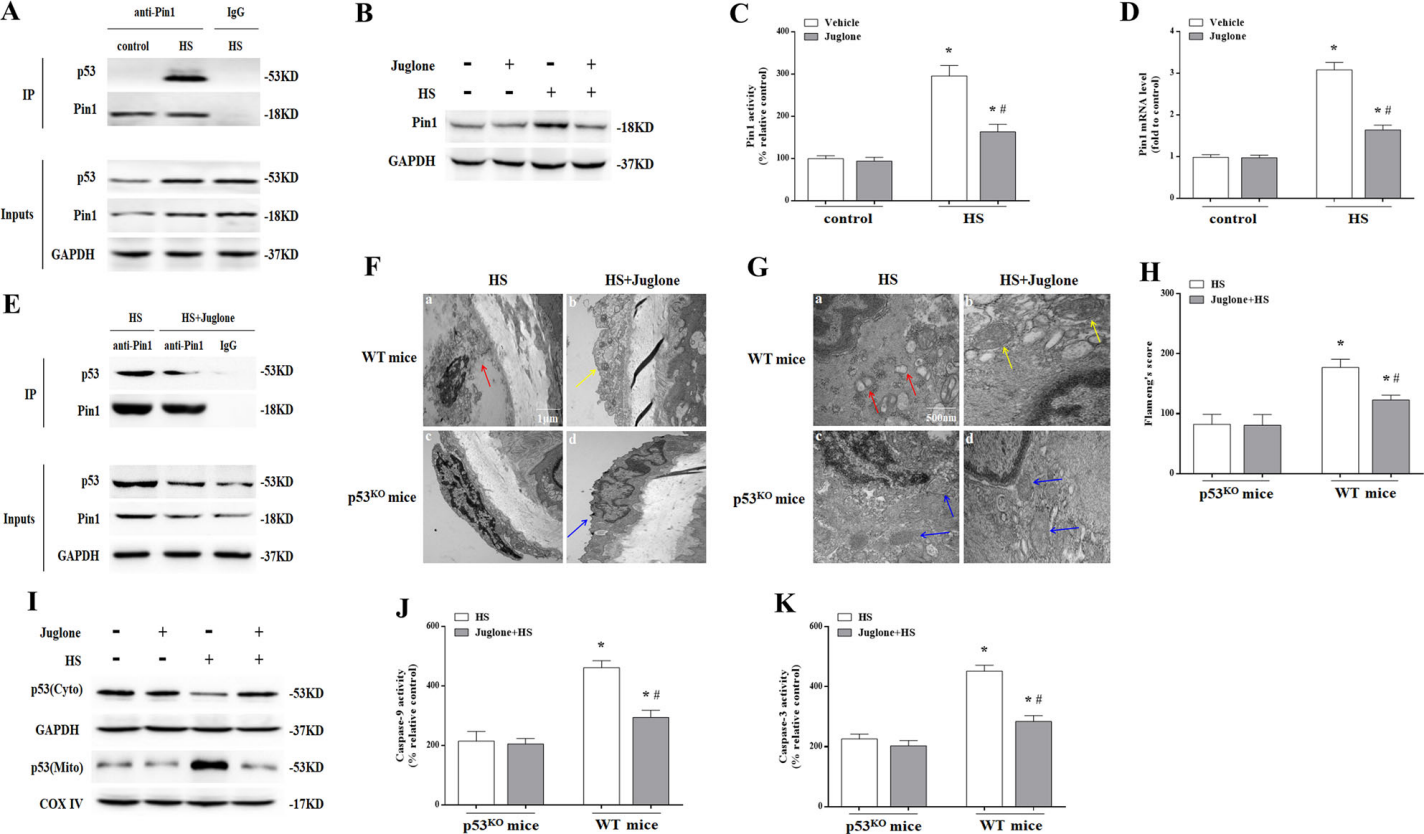
The effect of Pin1 inhibition on p53 transcription-independent pathway in aortic endothelium induced by heat stress (HS). Mice were put through the same HS assay as previously described. In the juglone-treated group, each mouse received an intraperitoneal injection of juglone (1 mg/kg body weight/d) for 3 consecutive days prior to exposure to HS. The mice were sacrificed at 6 h after exposure to HS and the aortic endothelium was isolated. **a** Interaction between endogenous p53 and Pin1 was analyzed by co-immunoprecipitation. IgG served as the negative control. **b** Pin1 protein levels were assessed by western blot. **c** Quantification of Pin1 activity in each group. **d** Quantification of Pin1 mRNA in each group. **e** Using co-immunoprecipitation to analyze the effect of Juglone on interaction between p53 and Pin1. IgG served as the negative control. **f** Aortic endothelium morphology was observed using transmission electron microscopy (TEM). **f**(a) Represents the HS group in wild-type mice and the red arrow indicates damaged endothelial cells; **f**(b) represents the HS + Juglone group in wild-type mice and the yellow arrow indicates damaged endothelial cells; **f**(c) represents the HS group in *p53*^KO^ mice; **f**(d) represents the HS + Juglone group in *p53*^KO^ mice and the blue arrow indicates damaged endothelial cells. **g** Mitochondrial morphology in endothelial cells of aortic endothelium was observed by TEM. **g**(a) Represents the HS group in wild-type mice and red arrows indicate mitochondria with severe damage, which appear swollen and irregularly shaped with disrupted and poorly defined cristae; **g**(b) represents the HS + Juglone group in wild-type mice and yellow arrows indicate mitochondria with partial damage, which appear mildly swollen and irregularly shaped and displayed less damage than the wild-type mice. **g**(c) and **g**(d) represent the *p53*^KO^ and *p53*^KO^ + Juglone group, respectively, and blue arrows indicate mitochondria with partial damage, which appear mildly swollen and irregularly shaped with no significant difference between the two groups, but less damage than in the wild-type mice after exposure to HS. **h** Mitochondria structural damage was evaluated by Flameng’s score. **i** Levels of p53 in mitochondrial and cytosolic fractions were assessed by western blot. **j** Enzymatic activity of Caspase-9 was measured using fluorogenic substrate Ac-LEHD-AFC. **k** Enzymatic activity of Caspase-3 was measured using fluorogenic substrate Ac-DEVD-AMC. ^*^P < 0.05 compared with the control group in *p53*^KO^ mice (**c**, **d**) or the HS group in *p53*^KO^ mice (**h**, **j**, **k**); ^#^*P* < 0.05 compared with the HS group in wild-type mice, *n* = 6

Meanwhile, HS also stimulated direct interactions between Pin1 and p53 in aortic endothelium as demonstrated by co-immunoprecipitation assays (Fig. 6a). Further in vivo studies showed pretreatment of wild-type mice with a Pin1-specific activity inhibitor, Juglone could significantly inhibit HS-induced Pin1 expression, enzymatic activity and mRNA level (Fig. 6b–d). What’s more, Juglone could impair HS-induced Pin1–p53 interaction in aortic endothelium (Fig. 6e). In addition, Juglone alleviated HS-induced endothelial cells damage (Fig. 6f(b)), prevented endothelial cells mitochondrial damage (Fig. 6g(b) and h), inhibited p53 mitochondrial translocation (Fig. 6i), and inhibited Caspase-9/3 activity in aortic endothelium in wild-type mice (Fig. 6j, k). However, Juglone had no effect on HS-induced endothelial cells damage (Fig. 6f(d), g(d) and h) and Caspase-9/3 activity (Fig. 6j, k) in *p53*^KO^ mice. Taken together, these data suggest that modification of p53 by Pin1 is an essential step for optimal execution of transcription-independent p53-mediated mitochondrial apoptosis after HS.

### Phosphorylation of p53 on Ser46 was an important event in Pin1 induction of p53 transcription-independent apoptotic activity

**Fig. 7:**
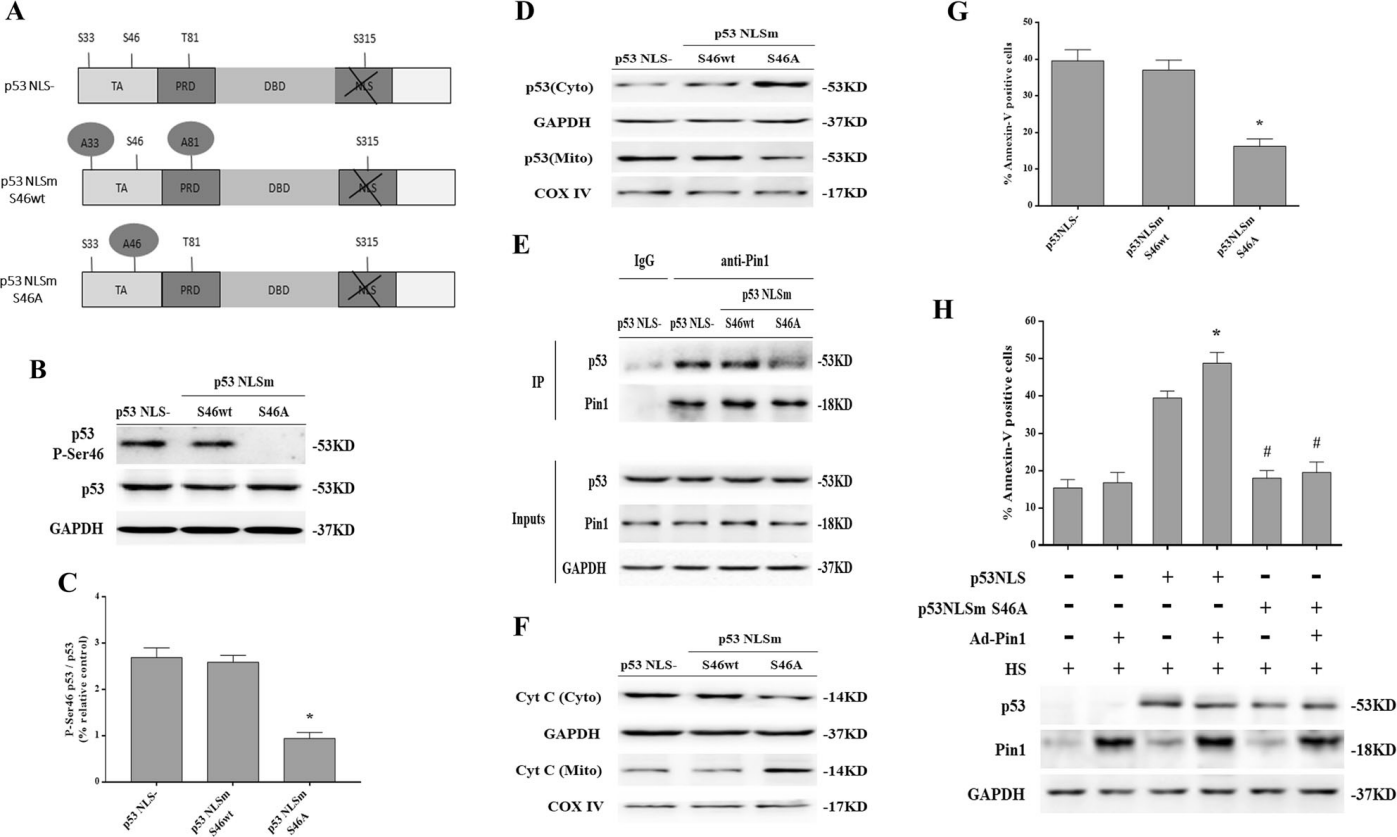
Phosphorylation of p53 on Ser46 was an important event in Pin1 induction of p53 transcription-independent apoptotic activity in response to heat stress (HS). H1299 *p53*^-/-^ cells were transfected with the p53NLS-, p53NLSm S46A, or p53NLSm S46wt constructs 48 h in advance. Cells were exposed to 43 °C for 2 h and then further incubated at 37 °C for 6 h. **a** Schematic of p53 indicating Pin1 consensus sites (phospho-Ser/Thr-Pro). TA represents transactivation domain, PRD represents proline enrichment domain, DBD represents DNA binding domain, and NLS represents nuclear localization signal. The p53NLS mutants had Ser/Thr-to-Ala substitutions at the Pin1 consensus sites at residue 46 (p53NLSm S46A) and two other major Pin1-binding sites at residues 33 and 81 ((p53NLSm S46wt). **b** Expression of P-Ser 46 p53 and p53 were determined by western blot. **c** Quantification of the P-Ser 46 p53 and p53 ratio. **d** Levels of p53 in the mitochondrial and cytosolic fractions were assessed by western blot. **e** Cell lysates normalized for p53 protein levels were subjected to co-immunoprecipitation to analyze interaction between endogenous p53 and Pin1. IgG served as the negative control. **f** The levels of Cyt C in the mitochondrial and cytosolic fraction were assessed by western blot. **g** Quantification of apoptosis in response to HS was performed with flow cytometry and Annexin V-FITC/PI staining. **h** H1299 *p53*^-/-^ cells were transfected with the p53 NLS- S46A construct 48 h and/or Ad-Pin1 24 h in advance. Cells were exposed to 43 °C for 2 h and then further incubated at 37 °C for 6 h. The levels of p53 and Pin1 proteins were measured by western blot. Apoptosis in response to HS was quantified by flow cytometry using Annexin V-FITC/PI staining. ^*^P < 0.05 compared with the HS group in H1299 p53NLS- cells; ^#^*P* < 0.05 compared with the HS group in Ad-Pin1 + H1299 p53NLS cells

Our above studies indicate that phosphorylation of p53 on Ser46 is triggered by HS. To determine whether phosphorylation of this or other sites is responsible for activating the p53 transcription-independent apoptotic activity induced by HS, we used cytosolic p53 constructs mimicking phosphorylation mutants with single and multiple substitutions of Ser/Thr with Ala residues at Pin1-binding sites (Fig. 7a). Expression of these constructs in H1299 *p53*^-/-^ cells demonstrated p53 Ser46 is required for HS-induced p53 transcription-independent apoptosis, because we employed a p53 mutant construct (p53NLSm S46A), which lacked a Pin1-binding sites (Ser46) in H1299 *p53*^-/-^ cells showed that p53NLSm Ser46A had no effect on p53 Ser46 phosphorylation (Fig. 7b, c); moreover, p53NLSm Ser46A inhibited p53 mitochondrial translocation (Fig. 7d), impaired Pin1-p53 interaction (Fig. 7e), reduced Cyt C release and apoptosis (Fig. 7f, g). By contrast, a p53 mutant (p53NLSm S46wt) that lacked the remaining two major Pin1-binding sites (Ser33 and Thr81) could efficiently induce p53 Ser46 phosphorylation (Fig. 7b, c); p53NLSm S46wt also promoted p53 binding to Pin1 and led to p53 transcription-independent apoptosis in response to HS (Fig. 7d–g). Importantly, the weak apoptotic activity of p53NLSm Ser46 could not be potentiated by overexpression of Pin1 (Fig. 7h). These results revealed that Ser46 phosphorylation is indeed the determinant for inducing mitochondrial apoptosis by a Pin1-dependent mechanism in response to HS.

### Pin1-mediated p53 transcription-independent apoptotic activity dependent on ROS (O_2_^−.^) production

**Fig. 8:**
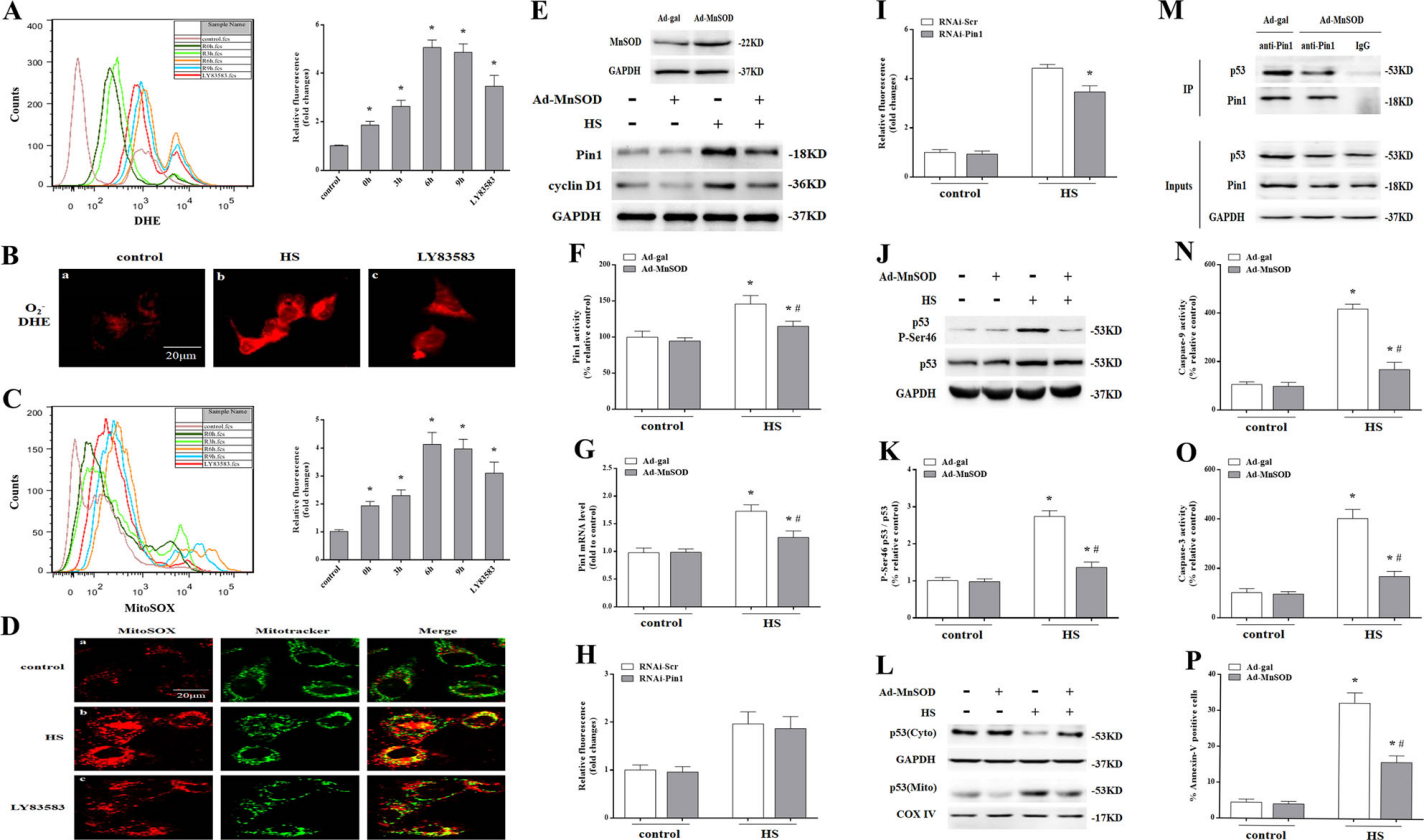
The role of reactive oxygen species (ROS) (O_2_^−.^) on Pin1-mediated p53 transcription-independent apoptotic activity after heat stress (HS). The *p53*^+/+^ mouse aortic endothelial cells (MAECs) were exposed to 43 °C for 2 h, then incubated at 37 °C for 0, 3, 6, or 9 h. LY83583 was used as a positive control for O_2_^−.^ (**a**–**d**). **a** ROS (O_2_^−.^) induced by HS was quantified by flow cytometry using dihydroethidium (DHE) staining. **b** Confocal laser scanning microscopy images of fluorescently labeled cells (red fluorescence). **c** Mitochondrial superoxide induced by HS was quantified by flow cytometry using MitoSOX staining. **d** Confocal laser scanning microscopy images of fluorescently labeled cells. The red fluorescence represents mitochondrial superoxide, green fluorescence represents Mito Tracker, orange fluorescence represents generation of mitochondrial superoxide. The *p53*^+/+^ MAECs were transfected with Ad-MnSOD 24 h in advance, exposed to 43 °C for 2 h, then incubated at 37 °C for 0 h (**e**–**g**). **e** Western blots of MnSOD protein expressed in transfected cells. Pin1 and cyclin D1 levels were measured by western blots. **f** Quantification of Pin1 activity. **g** Quantification of Pin1 mRNA. **h** The *p53*^+/+^ MAECs were transfected with Pin1 siRNA 48 h in advance, exposed to 43 °C for 2 h, then further at 37 °C for 0 h. Quantification of mitochondrial superoxide was analyzed by Flow cytometry. **i** The *p53*^+/+^ MAECs were transfected with Pin1 siRNA 48 h in advance, exposed to 43 °C for 2 h, then incubated at 37 °C for 6 h. Quantification of mitochondrial superoxide was analyzed by Flow cytometry. The HUVECs were transfected with Ad-MnSOD 24 h in advance, exposed to 43 °C for 2 h, then incubated at 37 °C for 6 h (**j**–**k**). **j** P-Ser 46 p53 and p53 levels were detected by western blot. **k** Quantification of the P-Ser 46 p53 and p53 ratio. The *p53*^+/+^ MAECs were transfected with Ad-MnSOD 24 h in advance, exposed to 43 °C for 2 h, then incubated at 37 °C for 6 h (**l**–**p**). **l** Intracellular location of p53 was determined by western blot. **m** Interaction between endogenous p53 and Pin1 was analyzed by co-immunoprecipitation. IgG served as the negative control. **n** Enzymatic activity of Caspase-9 was measured using fluorogenic substrate Ac-LEHD-AFC. **o** Enzymatic activity of Caspase-3 was measured using fluorogenic substrate Ac-DEVD-AMC. **p** Apoptosis induced by HS was quantified by flow cytometry using Annexin V-FITC/PI staining.^*^P < 0.05 compared with the control group (37 °C); ^#^*P* <0.05 compared with the HS group

**Fig. 9:**
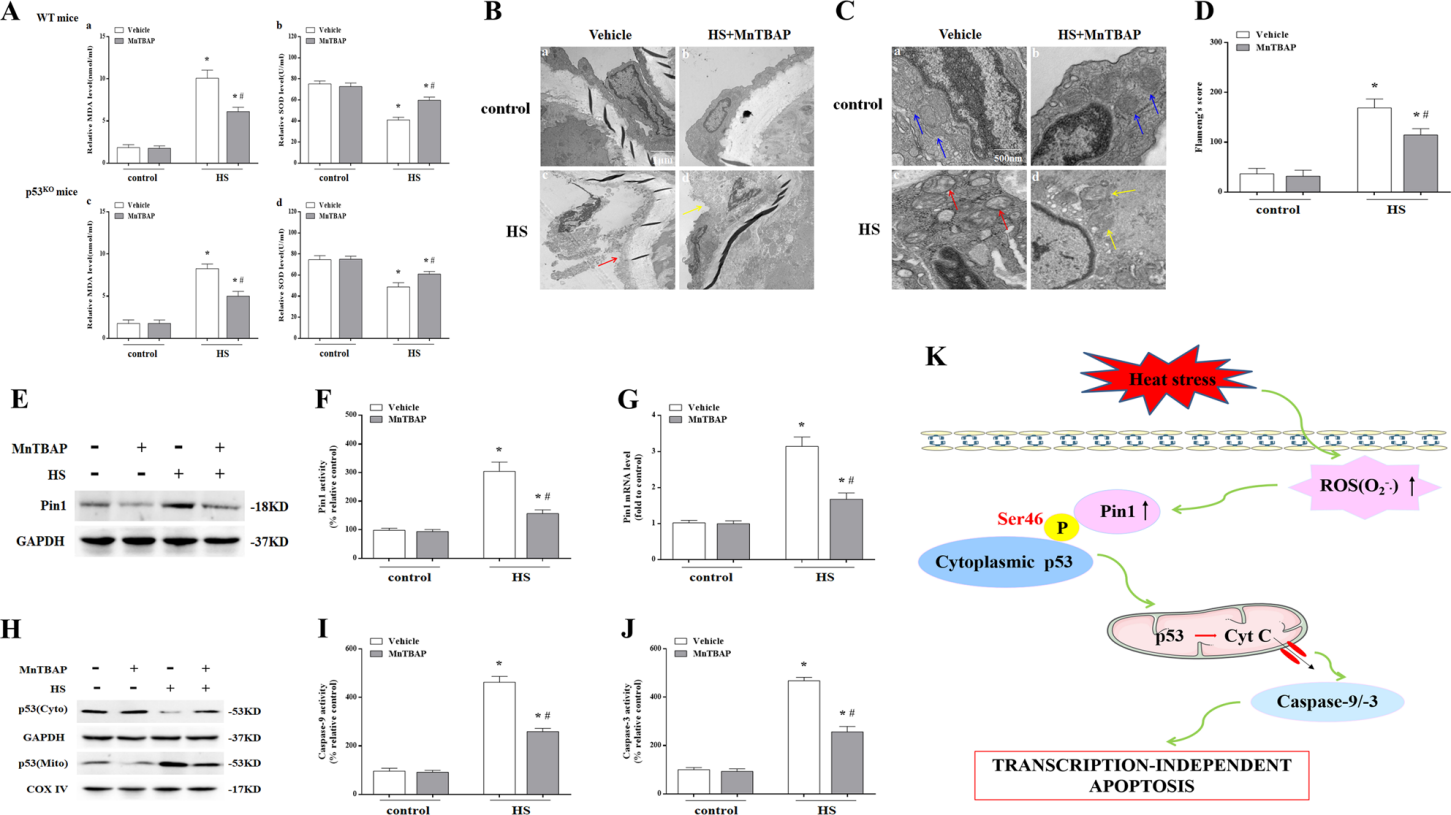
The effect of inhibition O_2_^−.^ on Pin1/p53 transcription-independent apoptosis pathways in aortic endothelium following exposure to heat stress (HS). Mice were put through the same HS assay as previously described. In the MnTBAP-treated group, each mouse received an intraperitoneal injection of MnTBAP (10 μg/g body weight) 1 h prior to HS. The mice were sacrificed at 6 h after exposure to HS and the aortic endothelium was isolated. **a** Quantification of malondialdehyde (MDA) and superoxide dismutase (SOD levels in wild-type and *p53*^KO^ mice. **b** Aortic endothelium morphology was observed by transmission electron microscopy (TEM) in wild-type mice. **b**(a) and **b**(b) represent the control and the control + MnTBAP group, respectively; **b**(c) represents the HS group and the red arrow indicates damaged endothelial cells; **b**(d) represents the HS + MnTBAP group and the yellow arrow indicates damaged endothelial cells. **c** Mitochondrial morphology in aortic endothelial cells was observed by TEM in wild-type mice. **c**(a) and **c**(b) represent the control and control + MnTBAP group, respectively, and blue arrows indicate mitochondrial morphology, which appeared normal with preserved membranes and cristae; **c**(c) represents the HS group and red arrows indicate mitochondria with severe damage, which appeared swollen and irregularly shaped with disrupted and poorly defined cristae; **c**(d) represents the HS + MnTBAP group and yellow arrows indicate mitochondria with partially damage, which appeared mildly swollen and irregularly shaped and had less damage than the wild-type mice exposed to HS. **d** Mitochondria structural damage was evaluated by Flameng’s score. **e** The levels of Pin1 protein were assessed by western blot. **f** Quantification of Pin1 activity in each group. **g** Quantification of Pin1 mRNA in each group. **h** The levels of p53 in the mitochondrial and cytosolic fraction were assessed by western blot. **i** Enzymatic activity of Caspase-9 was measured using fluorogenic substrate Ac-LEHD-AFC. **j** Enzymatic activity of Caspase-3 was measured using fluorogenic substrate Ac-DEVD-AMC. **k** ROS (O_2_^−.^) mediated HS-induced apoptosis in vascular endothelial cells through a Pin1/p53 transcription-independent pathway. ^*^P < 0.05 compared with the control group; ^#^*P* < 0.05 compared with the HS group, *n* = 6

Our previous studies showed HS resulted in intracellular ROS (O_2_^−.^ and H_2_O_2_) accumulation in HUVECs and O_2_^−.^ production began earliest and persisted^8,10^. Here, we detected intracellular ROS (O_2_^−.^) using the cell-permeable fluorescent dye dihydroethidium (DHE) and also observed an increase in a time-dependent manner in intracellular ROS (O_2_^−.^) production in *p53*^+/+^ MAECs after HS (Fig. 8a, b). Mito-SOX was used to detect mitochondrial ROS (O_2_^−.^) production. As shown in Fig. 8c, alterations in mitochondrial ROS (O_2_^−.^) production were consistent with dynamic changes in intracellular ROS (O_2_^−.^) accumulation. Image merging of Mito-Sox (red) with Mito tracker (green) further confirmed ROS (O_2_^−.^) generation from mitochondria was enhanced in *p53*^+/+^ MAECs after HS (Fig. 8d). Further in vivo experiments showed HS triggered a significant increase in malondialdehyde (MDA) concentration and decrease in superoxide dismutase (SOD) activity in aortic endothelium both of wild-type and *p53* ^KO^ mice (Fig. 9a). These data imply HS caused oxidative stress in aortic endothelial cells and endothelium, therefore, we hypothesize that oxidative stress may be involved in Pin1 induction of p53 transcription-independent apoptotic activity.

As upregulating MnSOD expression effectively reduces endothelial cell damage caused by oxidative stress^24^, we investigated whether mitochondrial ROS (O_2_^−.^) accumulation due to HS was also involved in Pin1-induced p53 transcription-independent apoptotic activity by infecting *p53*^+/+^ MAECs with MnSOD overexpression vector or control vector alone (control cells). Overexpression of MnSOD in *p53*^+/+^ MAECs significantly inhibited Pin1 and cyclin D1 proteins expression, Pin1 enzymatic activity and mRNA level at 0 h after HS (Fig. 8e–g). Meanwhile, using RNAi knockdown of Pin1, it has no effect on mitochondrial ROS production at 0 h after HS (Fig. 8h), whereas it could partially reduce mitochondrial ROS production at 6 h after HS in *p53*^+/+^ MAECs (Fig. 8i). In addition, overexpression of MnSOD could inhibit p53 Ser46 phosphorylation (Fig. 8j, k), p53 mitochondrial translocation (Fig. 8l), and interaction between Pin1 and p53 in endothelial cells (Fig. 8m). MnSOD overexpression caused a reduction in Caspase-9/-3 activity and apoptosis (Fig. 8n–p). In vivo experiments showed pretreatment with the O_2_^−.^ scavenger MnTBAP in mice, which decreased MDA level and increased SOD activity in aortic endothelium both of wild-type and *p53* ^KO^ mice (Fig. 9a). Pretreated with MnTBAP significantly alleviated HS-induced aortic endothelium damage (Fig. 9b(d)), promoted endothelial cell mitochondrial recovery in wild-type mice (Fig. 9c(d) and d). MnTBAP also decreased HS-induced Pin1 expression, enzymatic activity, and mRNA level (Fig. 9e–g), inhibited p53 mitochondrial translocation (Fig. 9h), as well as reduced Caspase-9/3 activity in aortic endothelium in wild-type mice (Fig. 9i–j). Overall, these results suggest HS led to upstream ROS generation, which was involved in Pin1 induction of p53 transcription-independent apoptotic activity.

## Discussion

In 2002, a new definition of heat stroke was introduced that suggested multi-organ system failure was due to the combined effects of heat cytotoxicity, coagulopathies, and systemic inflammatory response syndrome^6,7^. Notably, the primary event initiating thermal injury to vascular endothelium in HS patients is now considered multi-organ system dysfunction^5,7,25,26^. Previous studies performed in vitro and in vivo suggest vascular endothelial cells are early targets of thermal injury and these damaged cells are a notable factor in severe heat stroke^27,28^. Our recent studies also demonstrated HUVECs could be induced to undergo apoptosis during the acute phase of the response to HS^8,10^. Further in-depth research revealed HS-induced HUVEC apoptosis is associated with translocation of p53 to the mitochondria, which is mediated by ROS in a p53 transcription-independent apoptotic pathway^9,10^. However, the molecular mechanisms by which HS induces vascular endothelial cell apoptosis remain poorly understood. In particular, the intermediate mechanism of how ROS activate p53 transcription-independent apoptotic activity needs to be further explored.

The transcription factor p53 has pro-apoptotic activity through both transcription-dependent and -independent pathways^29^. When exposed to stress, p53 protein functions as a transcription factor to activate a number of genes, including *Noxa, Bax*, and *Puma*^29,30^. Furthermore, p53 also facilitates apoptosis via transcription-independent mechanisms, where it primarily acts by activating the mitochondria death pathway^31,32^. These two distinct pathways indicate there are two largely independent pools of p53 preexisting in the cytoplasm and nucleus of cells that, in response to genotoxic stress, simultaneously and rapidly stabilize^32^. Nuclear p53 export has previously been demonstrated to be a slow process lasting a minimum of 3–8 h^32,33^. By contrast, mitochondrial p53 translocation in response to stress is rapid, where it is detectable 30–60 min after exposure to death signal stimuli^32–34^. Following translocation of p53 to the mitochondria, mitochondrial dysfunction occurs that directly triggers mitochondrial outer membrane permeabilization and promotes the release of pro-apoptotic factors from the mitochondrial intermembrane space^34^. In sum, it is clear that during p53-mediated apoptosis, a fraction of p53 protein rapidly translocates to the mitochondria, where it triggers an early first wave of caspase-3 activation followed by an early wave of apoptosis^35,36^. This rapid first wave of apoptosis occurs via p53 transcription-independent mechanisms and precedes a second slower wave that is p53 transcription dependent^35^. In this study, we showed mitochondrial p53 translocation was evident at 3 h and peaked at 6-h exposure to 43 °C (HS). This was followed by mitochondrial apoptotic pathway activation, including mitochondrial membrane potential reduction, Cyt C release, Caspase-9/-3 activation, and early apoptosis in *p53*^+/+^ MAECs. However, HS-induced mitochondrial apoptotic pathway activation was significantly prevented in *p53*^-/-^ MAECs. In in vivo experiments, an absence of the *p53* gene alleviated injury to the aortic endothelium and endothelial cells, inhibited the HS-induced mitochondrial apoptosis pathway in aortic endothelium, and promoted survival. This shows that mitochondrial p53 translocation plays an essential role in HS-mediated apoptosis. To exclude the effect of p53 nuclear transcription on HS-induced apoptosis, we employed a nuclear import-deficient p53 construct (p53NLS-) in *p53*^-/-^ MAECs (p53NLS- MAECs). A significant amount of p53 translocation occurred in the mitochondria of p53NLS- MAECs, as well as increased Cyt C release, Caspase-9/-3 activity, and apoptosis following exposure to HS. Therefore, it is plausible that HS-induced mitochondrial p53 translocation that triggered mitochondrial apoptotic pathways both in vitro and in vivo. This is consistent with the notion that mitochondrial p53 can trigger fast apoptosis in response to amounts of damage.

Recent studies have led to significant discoveries concerning the influence of different partners and co-factors on a number of aspects of the p53 pathway^37^. Among these, Pin1, which is a prolyl-isomerase and phospho-specific transducer of post-translational modifications for several important signaling molecules, could be considered one of the most interesting^38^. Pin1 is essential for successful induction of the p53 apoptotic route in response to stress^16,19,20^. In particular, cis/trans isomerization of proline bonds is catalyzed by Pin1 following phosphorylation of serine or threonine residues (pSer/Thr-Pro), resulting in structural and functional alterations in cognate substrates in response to cues^19,38,39^. One of the main substrates of Pin1 is p53, which is phosphorylated by different kinases at Ser33, Ser46, Thr81, and Ser315 in response to severe stress, thus generating sites of recognition for Pin1 and notably influencing p53 stability and activity^16,17,19,20^. Although a crucial role for Pin1 in p53 transcription-dependent functions in the nucleus is strongly supported by several studies^16,17,40^, there are few studies published supporting a role for Pin1 in other growth-suppressive activities of p53, such as its mitochondrial death program. In the present study, HS-induced Pin1 expression, activity, and mRNA level both in wild-type and p53-deficient aortic endothelial cells and tissue. We observed HS-stimulated binding of Pin1 to p53 and Pin1 overexpression or knockdown strongly increased or reduced, respectively, mitochondrial translocation of p53 in HS-treated *p53*^+/+^ MAECs. Moreover, Pin1 overexpression or knockdown promoted or inhibited, respectively, HS-induced mitochondrial apoptotic pathway activation in H1299 p53NLS- cells.

In addition, Pin1 overexpression or knockdown had no effect on mitochondrial apoptotic pathway activation in p53-deficient cells following exposure to HS. Further studies found phosphorylation of Ser46 in p53 is a key event in Pin1-mediated p53 mitochondrial relocalization and function. A Ser46-Ala p53 mutant with a single substitution was unable to cause binding of Pin1 to p53 and p53 mitochondrial translocation promoted activation of the mitochondrial apoptosis pathway in response to HS. More interestingly, even Pin1 overexpression failed to allow apoptosis in a Ser46-Ala p53 mutant with a single substitution. Therefore, our findings imply phosphorylation of p53 on Ser46 is a crucial event in the pathway leading to induction of apoptosis by HS. It also indicates modification at this site is sufficient for Pin1 regulation of the apoptotic functions of p53.

Several reports have linked oxidative stress with HS and suggested synergistic augmentation of cell death as increased ROS generation was observed in heat-exposed cells^4,8,10,41^. Our previous data showed HS results in intracellular ROS (O_2_^−.^ and H_2_O_2_) accumulation in HUVECs, where O_2_^−.^ production begins earliest and persists^8,10^, and MnSOD overexpression effectively reduced endothelial cell damage caused by oxidative stress. Here, we found HS caused oxidative stress in aortic endothelium and promoted both intracellular and mitochondrial ROS (O_2_^−.^) production in *p53*^+/+^ MAECs. MnSOD overexpression inhibited Pin1-induced p53 transcription-independent apoptotic activity in *p53*^+/+^ MAECs after HS. In vivo experiments showed pretreating mice with an O_2_^−.^ Scavenger, MnTBAP, alleviated HS-induced endothelial cell damage and inhibited Caspase-9/3 activity in aortic endothelium. Altogether, ROS produced in response to acute HS may act as an upstream signal that triggers Pin1 induction of p53 transcription-independent pathways to promote the early wave of apoptosis.

Of note, our findings implied that p53 transcription-independent pathways could concur to HS-related toxicity, and the ability of Pin1 to potentiate the transcription-independent pathways of p53 might be critical in this respect. It suggested that interference with p53 mitochondrial translocation by targeting Pin1 might reduce HS-induced endothelial cells damage in patients. What’s more, since ROS as an upstream in Pin1/p53 signaling, indicating that preventing oxidative stress is crucial therapeutic intervention in patients with heat stroke. However, there are some problems with the present study. First, it has been shown that HS also induces activation of the mitochondrial apoptotic pathway in p53-deficient cells, although at much lower levels than in p53-sufficient cells. We suspect this may be related to cellular detection of damage from the heat or there may be other mechanisms involved in regulation of activation of the mitochondrial apoptotic pathway. Second, we found no difference in Pin1 expression and activity between p53-deficient and -sufficient cells and tissue, but Pin1 overexpression and knockdown had no effect on activation of the mitochondrial apoptotic pathway in p53-deficient cells after HS. It is unknown whether this is due to differences in Pin1 modification, conformation, or phenotype between p53-deficient and -sufficient cells and tissue following exposure to HS. Finally, our present study was limited to aortic endothelial cells and tissue, whereas endothelial cells in different tissues and organs have their own specific characteristics. Whether the mechanism of endothelial cell injury in the aorta is also applicable to other tissues and organs remains to be confirmed by further experiments.

## Conclusion

In conclusion (Fig. 9k), our results indicate that HS causes increased phosphorylation of p53 at Ser46, which leads to phosphorylation-dependent interactions between p53 and prolyl-isomerase Pin1 with consequent relocalization of cytosolic p53 to the mitochondria and activation of the mitochondrial apoptosis pathway. ROS were also found to be upstream signaling molecules in HS-induced Pin1/p53 signaling and involved in regulating activation of the mitochondrial apoptosis pathway. These findings have important implications for future mechanism-based therapeutic strategies in patients with heat stroke.

## Materials and methods

### Isolation of primary mouse endothelial cells

MAECs were isolated from wild-type and *p53*^KO^ mice using a collagenase perfusion protocol modified from Mika Kobayashi^42^. Mice were anesthetized with an intraperitoneal injection of pentobarbital sodium (10 mg/mL). The midline of the abdomen was incised and thorax opened to expose the heart and lungs. The abdominal aorta was cut in the middle to release the blood and then perfused from the left ventricle with 1 mL of phosphate-buffered saline (PBS) containing 1000 U/mL heparin. The aorta was dissected from the aortic arch to the abdominal aorta and immersed in Dulbecco’s modified Eagle's medium (DMEM) containing 20% fetal bovine serum (FBS) and 1000 U/mL of heparin. The fat and connecting tissue was rapidly removed with fine forceps under a stereoscopic microscope. A 24-gauge cannula was inserted into the proximal portion of the aorta. After ligation at the site with a silk thread, the inside of the lumen was briefly washed with serum-free DMEM. The other side was bound and filled with collagenase type II solution (2 mg/mL dissolved in serum-free DMEM). After incubating for 45 min at 37 °C, endothelial cells were removed from the aorta by flushing with 5 mL DMEM containing 20% FBS. Endothelial cells were collected by centrifuging at 1200 rpm for 5 min. The precipitate was then gently resuspended by pipetting with 2 mL of 20% FBS-DMEM and cultured in a 35-mm collagen type I-coated dish. To remove smooth muscle cells, the medium was removed after a 2-h incubation at 37 °C and the cells were washed with warmed PBS and cultured in medium 199 (GIBCO) supplemented with 10% FBS, 100 units/mL penicillin (Sigma), 100 mg/mL streptomycin (Sigma), 0.25 mg/mL amphotericin B (Sigma), 5 ng/mL endothelial growth factor (Boehringer Mannheim), and 1% l-glutamine (Sigma). One week later, confluent endothelial cells were seen. Cells were confirmed to be vascular endothelial cells based on morphology and immunohistochemical and immunofluorescence detection of the endothelial markers von Willebrand factor (vWF) and VE-Cadherin.

### Cell culture and treatments

The *p53*-null H1299 non-small cell lung carcinoma cell line was purchased from the Shanghai Institute of Cell Biology at the Chinese Academy of Sciences. H1299 cells were maintained in RPMI-1640 supplemented with 10% FBS. MAECs and H1299 cells were maintained in culture media in culture dishes, which were sealed with parafilm and immersed for 2 h in a circulating water bath thermoregulated at 37 ± 0.5 °C (control) or 43 ± 0.5 °C (HS)^8,9^. Culture medium was replaced with fresh medium and the cells were incubated at 37 °C for the indicated durations.

### Apoptosis assay

For cell cycle analysis, cells were either left untreated or exposed to 43 °C for 2 h before being analyzed by flow cytometry. Apoptosis was detected according to the Annexin V-FITC apoptosis detection kit manual (Invitrogen). Fluorescein isothiocyanate (FITC) is green-fluorescent dye which can conjugated to annexin V detecting apoptotic cells. Approximately 1 × 10^6^ cells were collected, washed with ice-cold PBS, and resuspended in binding buffer containing Annexin V-FITC. After a 10-min incubation in the dark at room temperature, the buffer was removed by centrifugation. Cells were resuspended in reaction buffer containing propidium iodide (PI) and then immediately analyzed by flow cytometry to measure apoptosis.

### Plasmid construction and stable transfection

The pcDNA3.1-p53NLS, pcDNA3.1-p53NLS S46A, pcDNA3.1-p53NLS M S46wt, and pcDNA3.1-Empty Vector constructs were synthesized by Cyagen Biosciences (Guangzhou, China). Cells were transfected with empty vector (pcDNA3.1) or the indicated construct (pcDNA3.1-p53NLS, pcDNA3.1-p53NLS S46A, or pcDNA3.1-p53NLS M S46wt) using Lipofectamine 2000. Culture medium containing G418 was used to select for stable transfectants. After incubating for 48 h at 37 °C in a humidified atmosphere with 5% CO_2_, the cells were exposed to HS for further experiments.

### Transfection and adenoviral infection

The Pin1 adenovirus (Ad-Pin1) was generated by Cyagen Biosciences (Guangzhou, China). For adenoviral infection of cells, approximately 5 × 10^6^ cells were trypsinized and resuspended in 500 μL serum-free DMEM. Ad-Pin1 (Multiplicity of Infection (MOI) = 10) was added to the cell suspension, which was then mixed. The tubes were incubated at 37 °C with 5% CO_2_ for 1 h with mixing every 10 min. Infected cells were then plated in 10-cm culture dishes containing 9.5 mL medium with serum and incubated for an additional 24 h. The cells were then exposed to HS for further experiments.

### Transfection with small-interfering RNA

Small-interfering RNA (siRNA) for p53 was designed and synthesized by Cyagen Biosciences (Guangzhou, China). The siRNA sequences for each gene and their corresponding scramble siRNA sequences are as follows: Pin1 sense, 5′-CGGGAGAGGAGGACUUUGA-3′ and Pin1 antisense, 5′-GCCAUUUGAAGACGCCUCG-3′; scrambled sense 5′-UGGUUUACAUGUCGACUAA-3′ and scrambled antisense, 5′-UUAGUCGACAUGUAAACCA-3′. Twenty-four hours prior to transfection, cells were plated in a six-well plate (Nest, Biotech, China) at 30–50% confluence and siRNAs were transfected into cells using TurboFectTM siRNA Transfection Reagent (Fermentas, Vilnius, Lithuania) according to the manufacturer’s protocol. After incubating for 48 h at 37 °C in a humidified atmosphere of 5% CO_2_, the cells were exposed to HS for further experiments.

### Measurements of ROS

After exposure to 43 °C (HS) for 2 h, the cells were further incubated at 37 °C for the indicated durations. Intracellular and mitochondrial ROS (O_2_^−.^) were detected using the fluorescent probes DHE (Molecular Probes) and MitoSOX (Molecular Probes), respectively. Cells were incubated with 1 µM DHE (red fluorescence) or 4 µM MitoSOX (red fluorescence) for 30 min at 37 °C in the dark. The fluorescence intensities of the intracellular and mitochondrial ROS (O_2_^−.^) probes were analyzed by flow cytometry and images were captured using laser scanning confocal microscopy.

### Mitochondrial membrane potential assay

The mitochondrial membrane potential was measured using the fluorescent probe JC-1 (Invitrogen, CA, USA). In mitochondria with normal membrane potentials, JC-1 forms aggregates that fluoresce red. In damaged and depolarized mitochondria, JC-1 forms monomers that fluoresce green. Cells were incubated in DMEM containing 5 µmol/L JC-1 for 15 min at 37 °C and images were captured using laser scanning confocal microscopy.

### Animals

Pathogen-free 6- to 8-week-old male C57BL/6 mice were purchased from the Experimental Animal Center of the Southern Medical University in Guangzhou, P.R. China [Certification: SCXK (Guangzhou) 2011–0015]. Pathogen-free 6- to 8-week-old male *p53* knockout (*p53*^KO^) mice were purchased from Bcgen (Biocytogen Co., Ltd) in Beijing, China. The animals were housed individually in controlled environmental conditions with a 12-h light/dark cycle and unrestricted access to pellet food and water throughout the study. All efforts were made to reduce the number of animals used and to minimize animal discomfort. The experimental protocols were approved by the Animal Care and Use Committee of the Southern Medical University, Guangzhou, China. None of the authors are members of this committee. The care of the animals was in accordance with the National Institutes of Health Guidelines, as well as with those of Chinese National.

### **Induction of HS**

Mice were fasted for 12 h prior to experiments, but were allowed water ad libitum. The animals in the HS group were placed in a pre-warmed incubator maintained at 35.5 ± 0.5 °C with a relative humidity of 60 ± 5% in the absence of food and water. The rectal core temperature (Tc) was continuously monitored with a rectal thermometer until the Tc reached 42 °C. The animals in the control group were sham heated at a temperature of 25 ± 0.5 °C and a humidity of 35 ± 5% for a time comparable to that of the HS group^43^. The mice were sacrificed at the indicated times after HS and the aortic endothelium was isolated.

### Mitochondrial fractionation

Briefly, ∼150 mg of aortic endothelium tissue was minced or cells were grown on 100 mm plates until 80–90% confluency, washed twice with ice-cold PBS, scraped into ice-cold PBS, followed by centrifugation at 1000 *g* for 5 min at 4 °C. Cell pellets were resuspended in mitochondria isolation buffer (5 mM Hepes (pH7.4), 3 mM MgCl_2_, 1 mM EGTA, and 250 mM sucrose) containing protease and phosphatase inhibitors (10 μg/mL aprotinin, 10 μg/mL pepstatin A, 10 μg/mL leupeptin, 1 mM phenylmethanesulfonyl fluoride (PMSF), 2 mM sodium orthovanadate, 5 mM sodium fluoride). Lysates were passed through a 25-gauge 5/8 needle 20 times using a 1 mL syringe and centrifuged at 1000 *g* for 20 min. Supernatants were collected and cytosolic extracts were recovered by centrifuging at 10,000 *g* for 15 min. The pellets were crude mitochondria and lysed with SDS sample buffer (63 mM Tris-HCl, 10% glycerol, and 2% SDS) containing protease and phosphatase inhibitors before western blotting.

### Nuclear and cytosolic fractionation

Nuclear and cytosolic fractions were prepared from freshly isolated aortic endothelium tissue or cells using a commercially available nuclear extraction kit (Beyotime Institute of Biotechnology, China). In all, ∼75 mg of aortic endothelium tissue was minced or cells were grown on 100 mm plates until 80–90% confluency, then homogenized in Cytoplasmic Extraction Reagent I buffer containing protease inhibitor cocktail Complete, EDTA-free. After a series of wash steps, nuclear proteins were extracted in high salt Nuclear Extraction Reagent buffer supplemented with protease inhibitors. The cytosolic fraction was spun at 100,000 *g* at 4 °C for 60 min to obtain a pure cytosolic fraction.

### Western blot analysis

The protein concentrations of extracts were determined using an Enhanced BCA Protein Assay Kit (Beyotime Institute of Biotechnology, China). Western blot analysis was performed as described previously using p53, Pin1, Cyt C, and MnSOD antibodies (1:1000; Abcam or Cell Signaling Technology). A horseradish peroxidase-conjugated anti-rabbit or anti-mouse IgG antibody was used as the secondary antibody (Zhongshan Inc., China) and signal was visualized using enhanced chemiluminescence (Pierce, Rockford, IL, USA).

### Co-immunoprecipitation

After exposure to HS, cells or aortic endothelium were harvested for the preparation of whole-cell or tissue lysates using RIPA buffer (10 mM Tris-HCl pH 8, 2 mM EDTA, 0.1% SDS, 0.1% sodium deoxycholate, 140 mM NaCl, and 1 × Triton and supplemented with 1 mM phenylmethylsulfonyl fluoride and protease inhibitors; Sigma). Cells or tissue lysates were incubated on ice for 20 min, vortexed, and centrifuged at 6600 *g* for 10 min to remove cellular debris. Protein concentrations were determined with Bradford Reagent (Sigma). Three milligrams of cell or tissue lysate was incubated overnight at 4 °C with 3 mg of anti-p53. The immunocomplexes were collected by incubating with a mix of Protein A-Agarose and Protein G-Sepharose (Sigma) overnight at 48 °C. The beads were washed three times: the first wash was with RIPA buffer and the remaining two with PBS. The beads were then resuspended in 2X Lysis buffer, loaded directly onto a 10% SDS-polyacrylamide gel, and then subjected to western blot with the indicated antibodies. IgG served as the negative control.

### Pin1 activity assay

Pin1 activity assay from all treatment groups using a Pin1 activity assay kit (ANASPEC, USA) following the manufacturer’s protocols. Briefly, cells or aortic endothelium tissue were lysed by sonication in lysis buffer (50 mM N-2-hydroxyethylpiperazine-N’-2-ethanesulfonic acid, 100 mM NaCl, 0.25% 3-[(3-cholamidopropyl) dimethylammonio]-1-propanesulfonate (CHAPS), 5 mM NaF, 1 mM β-glycerophosphate, and 1 mM ethylene glycol tetraacetic acid) at 4 °C. Then prepared working solutions, including Pin1 substrate solution, recombinant Pin1 diluent, Pin1 inhibitor, reaction developer. The reaction was started by adding 50 μl of Pin1 substrate solution into each well. For best accuracy, it is advisable to have the substrate solution equilibrated to the assay temperature. Mix reagents completely by shaking the plate gently for no >30 s. Measure fluorescence signal: for kinetic reading: immediately start measuring fluorescence intensity at Ex/Em = 490 nm/520 nm continuously and record data every 5 min for 60–120 min. For end-point reading: incubate the reaction at room temperature for 60–120 min. Keep plate away from direct light, then measure fluorescence intensity at Ex/Em = 490 nm/520 nm.

### Quantitative real-time PCR (qRT–PCR) analysis

The total RNA was isolated using Sepasol reagent (Nakalai Tesche, Kyoto, Japan). First-strand complementary DNAs were synthesized using PrimeScript reverse transcriptase with oligo (dT). The SYBR Green real-time PCR amplifications (Invitrogen) were conducted with MiniOpticon real-time PCR detection system (Bio-Rad Laboratories Inc., Hercules, CA, USA). The following primer sequences were used: Pin1 5′-TCGGGAGAGAGGAGGACTTTG-3′ (sense) and 5′-GGAGGATGATGTGGATGCC-3′ (antisense); GAPDH 5′-AGATCCACAACGGATACATT-3′ (sense) and 5′-TCCCTCAAGATTGTCAGCAA-3′ (antisense).

### Caspase activity assay

After exposure to HS, cells or aortic endothelium were harvested, lysed, and cell or tissue lysates were incubated at –80 °C for 30 min prior to incubation with the appropriate caspase substrates at 37 °C using a Quadruple Monochromator Microplate Reader (Infinite M1000, Tecan US, NC, USA). Caspase activity was measured based on cleavage of the fluorogenic peptide substrate Ac-LEHD-AFC for Caspase-9 and Ac-DEVD-AMC for Caspase-3. Caspase activity is represented as relative cumulative fluorescence of the kinetic reaction relative to untreated controls.

### Morphological observation

The morphological changes in the aortic endothelium and mitochondria in the endothelial cells were observed by TEM. The aortic endothelium was fixed with 2.5% glutaraldehyde and stained with cacodylate-buffered osmium tetroxide (OsO4). Sections were prepared and examined under an electron microscope (Philips CM10; Philips, Eindhoven, The Netherlands).

### Statistical analysis

All data were analyzed for statistical significance using SPSS 13.0 software (SPSS, Chicago, IL, USA). Data are from at least three independent experiments performed in duplicate and are expressed as mean ± SD. Statistical comparisons of the results were performed using one-way analysis of variance (ANOVA). A *P* < 0.05 was considered to be statistically significant.

## Data Availability

The data that support the findings of this study are available from the corresponding author on reasonable request.
